# *Porphyromonas gingivalis* ATCC 33277 promotes intercellular adhesion molecule-1 expression in endothelial cells and monocyte-endothelial cell adhesion through macrophage migration inhibitory factor

**DOI:** 10.1186/s12866-018-1156-1

**Published:** 2018-02-26

**Authors:** Wanyue Xu, Yaping Pan, Qiufang Xu, Yun Wu, Jiayu Pan, Jingya Hou, Li Lin, Xiaolin Tang, Chen Li, Jingbo Liu, Dongmei Zhang

**Affiliations:** Department of Periodontics and Oral Biology, School of Stomatology, China Medical University, Nanjing North St.117, Shenyang, Liaoning 110002 China

**Keywords:** *Porphyromonas gingivalis*, Macrophage migration inhibitory factor, Intercellular cell adhesion molecule-1, Endothelial cells

## Abstract

**Background:**

*Porphyromonas gingivalis* (*P. gingivalis*), one of the main pathogenic bacteria involved in periodontitis, induces the expression of intercellular adhesion molecule − 1 (ICAM-1) and monocyte-endothelial cell adhesion. This effect plays a pivotal role in atherosclerosis development. Macrophage migration inhibitory factor (MIF) is a multifunctional cytokine and critically affects atherosclerosis pathogenesis. In this study, we tested the involvement of MIF in the *P. gingivalis* ATCC 33277-enhanced adhesive properties of endothelial cells.

**Results:**

Endothelial MIF expression was enhanced by *P. gingivalis* ATCC 33277 infection. The MIF inhibitor ISO-1 inhibited ICAM-1 production in endothelial cells, and monocyte-endothelial cell adhesion was induced by *P. gingivalis* ATCC 33277 infection. However, the addition of exogenous human recombinant MIF to *P. gingivalis* ATCC 33277-infected endothelial cells facilitated monocyte recruitment by promoting ICAM-1 expression in endothelial cells.

**Conclusions:**

These experiments revealed that MIF in endothelial cells participates in the pro-atherosclerotic lesion formation caused by *P. gingivalis* ATCC 33277 infection. Our novel findings identify a more detailed pathological role of *P. gingivalis* ATCC 33277 in atherosclerosis.

## Background

Many epidemiological studies have associated severe forms of periodontitis with atherosclerosis [1]. *Porphyromonas gingivalis* (*P. gingivalis*), a Gram-negative oral anaerobe, has been identified as one of the main pathogenic bacteria in periodontitis [2]. The DNA of *P. gingivalis* has been found in coronary stenotic artery plaques of myocardial infarction patients [3, 4]. Furthermore, animal experiments have shown that *P. gingivalis* infection directly induces and accelerates atherosclerotic lesion development in pigs and mice [5, 6]. In vivo studies have suggested that *P. gingivalis* enters the systemic circulation through inflammation-injured epithelial structures; then, this bacterium adheres to and invades vascular endothelial cells, proliferates in host cells, promotes the release of a variety of proinflammatory cytokines and induces atherosclerosis formation [7–11].

Macrophage migration inhibitory factor (MIF) has been recognized as a key factor in the vascular processes leading to atherosclerosis [12–14]. MIF expression in endothelial cells is dysregulated in response to proatherogenic stimuli during the development of atherosclerotic lesions in humans, rabbits, and mice [15, 16]. Recent research showed that MIF increased monocyte recruitment during the process of atherosclerosis development [17]. One of the mechanisms of this effect is the MIF-mediated up-regulation of adhesion molecule expression in vascular endothelial cells, which causes the monocytes flowing rapidly in blood circulation to decelerate, roll on the vessel wall, aggregate and adhere to the vessel wall [18].

Studies have shown that increased intercellular adhesion molecule − 1 (ICAM-1) expression is one of the molecular mechanisms of the pathological changes during the early stage of atherosclerosis. By mediating leukocyte adhesion, ICAM-1 increased plaque instability and accelerated plaque rupture and thrombosis, resulting in cardiovascular disease (CVD) events [19].

Our previous studies have found that *P. gingivalis* infection increases ICAM-1 expression in endothelial cells and monocyte-endothelial cell adhesion [20]. These findings suggested that *P. gingivalis* induces the inflammatory process of atherosclerosis. However, the exact role that *P. gingivalis* plays in the development of atherosclerosis is still unclear. We hypothesized that *P. gingivalis* infection promotes the formation of atherosclerosis through MIF. In the present study, we examined the MIF production induced by *P. gingivalis* ATCC 33277 in endothelial cells. We also investigated the impact of MIF on the adhesive properties of endothelial cells pretreated with the antagonist ISO-1 or human recombinant MIF (rMIF) plus ISO-1. Our novel findings have identified a more detailed pathological role of *P. gingivalis* in atherosclerosis.

## Methods

### Bacterial strains and culture methods

The *P. gingivalis* strain ATCC 33277 was anaerobically (80% N_2_, 10% O_2_, 10% H_2_) cultured in brain heart infusion broth that contained defibrinated sheep’s blood (5%), hemin (0.5%) and vitamin K (0.1%) at 37 °C. Bacterial cells were cultured overnight until the optical density reached 1.0 at 600 nm; then, the cells were resuspended in Dulbecco’s modified Eagle medium (DMEM, Gibco BRL, Carlsbad, CA, USA) at a final concentration of 1 × 10^12^ cells/L.

### Cell lines

The human umbilical vein endothelial cell line EA.hy926 and the THP-1 monocyte model (a monocytic leukaemia cell line) were purchased from Keygen Biotech company (Nanjing, China). EA.hy926 cells were cultured in DMEM containing 15% fetal bovine serum, and the THP-1 cells were cultured in DMEM containing 10% fetal bovine serum at 37 °C in 5% CO_2_. EA.hy926 cells (10^5^ cells mL^− 1^) were seeded in the tissue plate wells and were cultured until a confluent monolayer formed for subsequent study. Cell viability, which was > 90% for all the infection assays, was determined by trypan blue exclusion assay. THP-1 cells were labeled with the fluorescent dye calcein AM (0.1 mg/mL; BioVision, CA, USA) for 30 min before being co-cultured with EA.hy926 cells.

### Enzyme linked immunosorbent assay (ELISA)

Bacterial suspensions were added to the EA.hy926 cells at a multiplicity of infection (MOI) of 100 for 4, 10 or 24 h, while *Escherichia coli* (*E. coli*) lipopolysaccharide (LPS) (1 μg/mL; Cayman Chemical, Ann Arbor, MI, USA) was used as a positive control [21]. The MIF level was determined using ELISA kits (BD Biosciences, Mountain View, CA, USA). The optical density was measured at 450 nm, and the MIF concentration was extrapolated from the standard curve according to the manufacturer’s instructions.

### Western blot

The EA.hy926 cells were pretreated with the MIF antagonist ISO-1 (25 μM; Cayman Chemical) or human rMIF (0.5 μg/mL; Cayman Chemica) plus ISO-1 for 1 h; then, the cells were infected with *P. gingivalis* ATCC 33277 at an MOI of 100 for 24 h. The whole cell protein of EA.hy926 cells was extracted, and Western blotting was performed.

The EA.hy926 cells were lysed, and the protein concentration was determined by a BCA assay. Equal amounts of whole cell lysate were separated with 8% SDS-polyacrylamide gel electrophoresis and were transferred to a nitrocellulose filter membrane. After blocking, the protein was blotted with rabbit monoclonal anti-ICAM-1 antibody (1:500; Wanlei, Shenyang, China) and goat anti-rabbit Dylight 800-conjugated fluorescent antibody (1:1000; Abbkine Inc., Redlands, CA, USA). Western blot analysis was performed with Odyssey CLX (LI-COR, Lincoln, NE, USA).

### Quantitative real-time polymerase chain reaction (qRT-PCR)

EA.hy926 cells were treated as mentioned above (in Western blot analysis). Then, the total RNA of EA.hy926 cells was extracted using TRIzol reagent (Invitrogen, Carlsbad, CA, USA). To remove the genomic DNA, total RNA was treated with DNase I for 2 min at 42 °C following the manufacturer’s protocol. The RNA integrity was checked via electrophoresis on 1.0% agarose gels. The RNA purity was identified by the 260/280 nm optical density ratio, and RNA samples with an 260/280 nm optical density ratio greater than 1.9 were selected for later analysis. Next, cDNA was synthesized using a reverse transcription system (Vazyme, Beijing, China) [22].

qRT-PCR was performed using Biosystems 7500 Fast real-time PCR and SYBR Premix Ex Taq II (RR047, RR420, Takara, Tokyo, Japan) according to the manufacturer’s instructions. Specific primers were 5’-TCGGCACAAAAGCACTATATG -3′ (forward), 5’-ACAGGACAAGAGGACAAGGC-3′ (reverse) for ICAM-1 and 5’-GAAGGTCGGAGTCAACGGAT-3′ (forward) and 5′- CCTGGAAGATGGTGATGG GAT-3′ (reverse) for Glyceraldehyde-3-phosphate dehydrogenase (GAPDH). Primers were designed with Primer Premier 5 software. The results are expressed as the relative ICAM-1 mRNA levels compared with the untreated control, which was considered 100%.

### THP-1 adhesion to EA.hy926 cells

EA.hy926 (10^5^ cells mL^− 1^) were seeded on 6-well plates at 2 × 10^5^ cells per well and were cultured to form a confluent monolayer. Then, the cells were pretreated with the MIF antagonist ISO-1 (25 μM) or rMIF (0.5 μg/mL) plus ISO-1 for 1 h. Next, the cells were infected with *P. gingivalis* ATCC 33277 at an MOI of 100 for 23 h. Next, 1 × 10^6^ THP-1 cells were labelled with 5 μM calcein-AM and were co-cultured with the EA.hy926 cells for another 1 h. Non-adherent THP-1 cells were gently washed away with PBS twice. The adherent THP-1 cells remaining on the monolayer of endothelial cells were visualized using a fluorescence microscope (Nikon 80i, Tokyo, Japan); 3 fields under the microscope (× 100) were randomly selected; and the fluorescence-labeled THP-1 cells were assessed by cell counting assays [23].

All the experiments were performed in triplicate wells for each condition and repeated at least three times.

### Statistical analysis

All data are presented as the means ± SD of three independent experiments. Statistical analysis was performed using one-way ANOVA, and the Student-Newman-Keul test was applied to compare differences from each other group (SPSS 17.0 software, IBM). *P*-values < 0.05 were considered statistically significant.

## Results

### *P. gingivalis* ATCC 33277 infection enhances MIF secretion in EA.hy926 cells

**Fig. 1:**
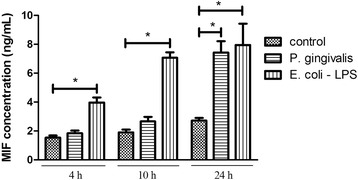
*Porphyromonas gingivalis* (*P. gingivalis*) ATCC 33277 infection enhances MIF secretion in EA.hy926 cells*.* EA.hy926 were challenged with *Escherichia coli* (*E. coli*) lipopolysaccharide (LPS, 1 μg/mL) or *P. gingivalis* (MOI = 100) for 4, 10 or 24 h. MIF levels was analyzed using ELISA. Infection of *P. gingivalis* significantly increased MIF secretion in EA.hy926 cells. EA.hy926 were incubated with medium only as a control. E.coli LPS was used as a positive control. The I bar shows the standard deviation. *P < 0.01

We evaluated the effect of *P. gingivalis* ATCC 33277 on MIF expression. *E. coli*-LPS was used as a positive control, since MIF release is induced by proinflammatory factors such as LPS [21, 24]. The ELISA results revealed that *P. gingivalis* ATCC 33277 infection significantly increased MIF secretion in EA.hy926 cells. Compared with the control level, MIF expression was increased 2.25-fold (MOI = 100) by *P. gingivalis* ATCC 33277 infection for 24 h (*P* < 0.01). *P. gingivalis* ATCC 33277 did not significantly affect MIF expression at the early time point, including 4 and 10 h (Fig. 1). *P. gingivalis* infection at an MOI of 100 for 24 h was chosen to evaluate the impact of MIF on the increased adhesive properties of endothelial cells in the following studies.

### *P. gingivalis* ATCC 33277 infection increase expression of ICAM-1 and ICAM-1 mRNA in EA.hy926 through MIF

**Fig. 2:**
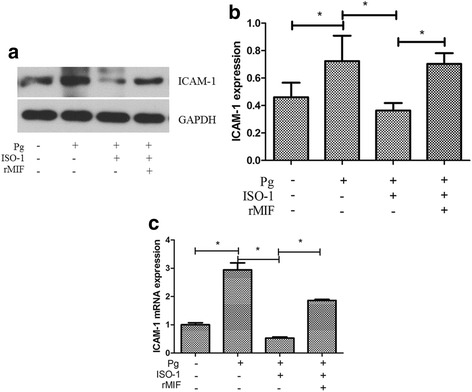
Macrophage migration-inhibitory factor (MIF) acted as a regulator in *Porphyromonas gingivalis* (*P. gingivalis*)-induced intercellular adhesion molecule-1 (ICAM-1) expression. **a** Western blot analysis of ICAM-1 in EA.hy926 cells. **b** Quantitative analyses of the optical density relative to the internal reference protein GAPDH in Western blot analysis. **c** Quantitative Real-time PCR analysis of ICAM-1 mRNA. Infection with *P. gingivalis* ATCC 33277 (MOI = 100, 24 h) could induce the expression of ICAM-1 and ICAM-1 mRNA in EA.hy926 cells, this inductive effect of *P. gingivalis* was blocked by the MIF antagonist ISO-1. Sufficient exogenous recombinant MIF (rMIF) supplementation rescued the ICAM-1 expression. Data are presented as means ± SD from three independent experiments. * *P* < 0.01

To determine the impact of MIF on ICAM-1 expression, the MIF antagonist ISO-1 and rMIF were used. The results revealed that *P. gingivalis* ATCC 33277 infection (MOI = 100:1, 24 h) induced a significant increase in ICAM-1 expression. We discovered that this inductive effect of *P. gingivalis* ATCC 33277 was blocked by the MIF antagonist ISO-1. *P. gingivalis*-induced ICAM-1 expression was significantly reduced (by 49.78%) by ISO-1. Moreover, the inhibitory effect of ISO-1 was neutralized by exogenous rMIF. Sufficient exogenous rMIF supplementation rescued ICAM-1 expression. ICAM-1 expression was increased 1.95-fold in the rMIF group compared to the ISO group (Fig. 2a and b).

These findings were further confirmed by the qRT-PCR results, which detected the *ICAM-1* gene transcription level under the same conditions as described above. The ICAM-1 mRNA level was also significantly reduced in ISO-1-treated cells, with an 81.97% reduction compared with that in *P. gingivalis*-infected cells. Similarly, exogenous rMIF increased the ICAM-1 mRNA level, which was 3.51-fold higher in the rMIF group compared with the ISO group (Fig. 2c).

### MIF regulates the increased monocyte adhesion to endothelial cells infected with *P. gingivalis* ATCC 33277

**Fig. 3:**
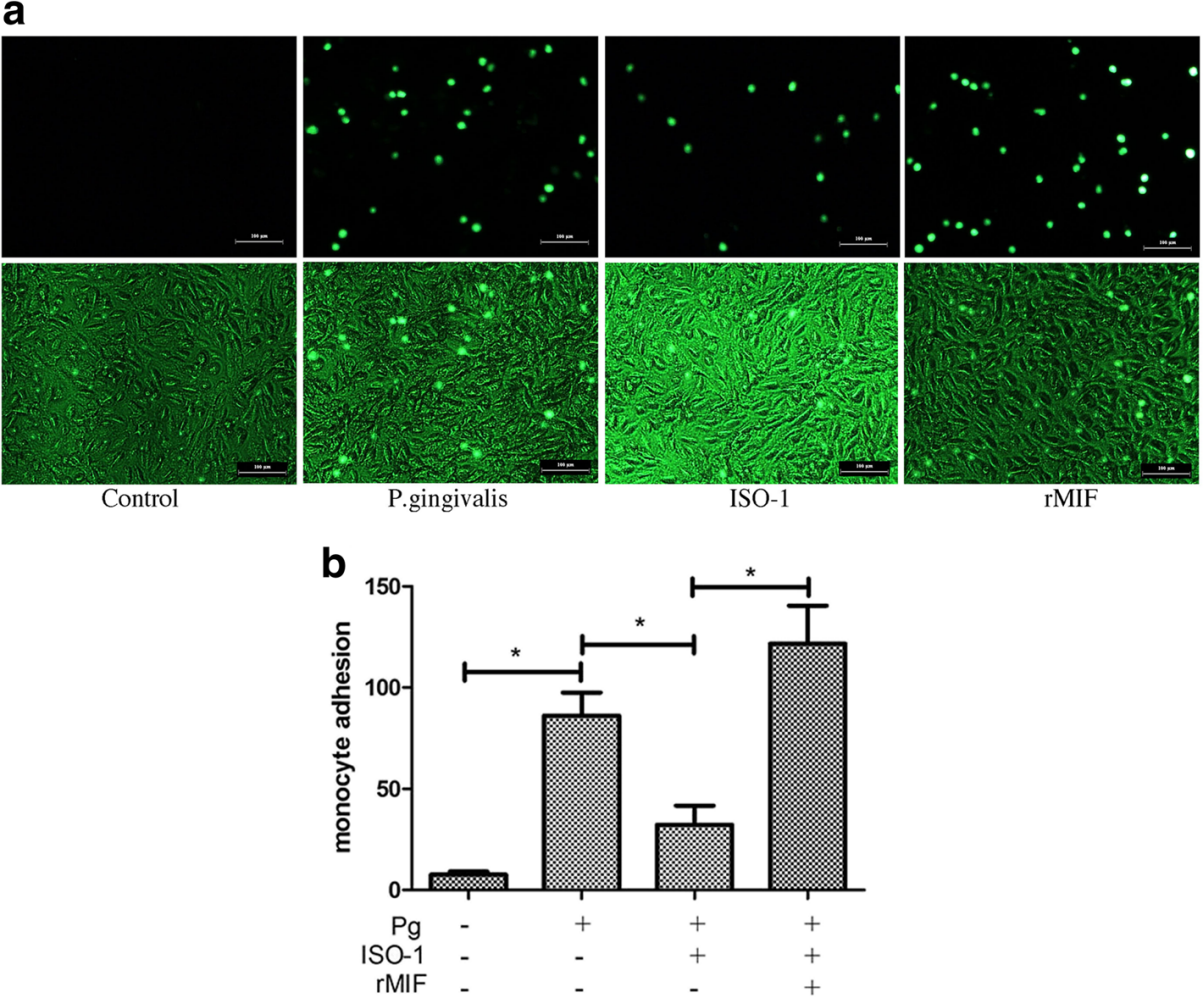
Macrophage migration-inhibitory factor (MIF) regulated the increased monocyte adhesion to endothelial cells infected with *Porphyromonas gingivalis* (*P. gingivalis*)*.* **a** THP-1 cell adhesion to EA.hy926 cells was labeled by calcein-AM and visualized. Pictures are representative fields captured by flurencence microscope (upper line) or microscope (lower line) of three independent experiments (magnification × 100). **b** The histogram of the evaluation of adhered THP-1 cell assessed by cell count assay. Compared with uninfected cells, *P. gingivalis* ATCC 33277 infection (MOI = 100, 24 h) markedly increased THP-1 cell adhesion to endothelial cells (*P* < 0.01). In contrast, cell adhesion was decreased in ISO-1-treated cells compared with those infected with *P. gingivalis* ATCC 33277 (*P* < 0.01). And THP-1 cell adhesion to EA.hy926 cells was recovered by exogenous rMIF addition. * *P* < 0.01. Scale bar = 100 μm

To investigate the role of MIF in *P. gingivalis*-induced monocyte-endothelial cell adhesion, we used the fluorescent dye calcein-AM to highlight the adhesive THP-1 cells. THP-1 cell adhesion to EA.hy926 cells was visualized using fluorescence microscopy. The adhesion experiment results were consistent with ICAM-1 expression. Compared with uninfected cells, *P. gingivalis* ATCC 33277 infection (MOI = 100, 24 h) markedly increased THP-1 cell adhesion to endothelial cells (*P* < 0.01). In contrast, cell adhesion was decreased in ISO-1-treated cells compared with those infected with *P. gingivalis* ATCC 33277 (*P* < 0.01). In addition, THP-1 cell adhesion to EA.hy926 cells was recovered by exogenous rMIF addition, as shown in Fig. 3.

These results, in combination with those in Fig. 2, suggested that expression of ICAM-1 in endothelial cells and monocyte-endothelial cell adhesion caused by *P. gingivalis* ATCC 33277 infection could be regulated by MIF.

## Discussion

Numerous cross-sectional, case-control and cohort epidemiological studies suggest that periodontal infection is associated with atherosclerotic CVD, independent of confounding factors such as smoking and obesity [25–27], and systemic inflammation has been proposed as a possible mediator [24], which ultimately enhances the adherence of circulating monocytes to vascular endothelial cells. Our prior study found that *P. gingivalis* infection induced ICAM-1 expression and monocyte recruitment, which are crucial events leading to atherosclerosis pathogenesis [20]. This result is consistent with the findings of Velsko [10]. *P. gingivalis* is believed to play a pivotal role in the development of atherosclerosis.

MIF is a proinflammatory cytokine that plays a critical role in the initiation and progression of chronic inflammatory and immune-mediated diseases such as atherosclerosis [28]. Under normal circumstances, the MIF protein level is very low. However, in atherosclerotic lesions, MIF is secreted in large quantities by vascular endothelial cells, and a relatively small amount of MIF is released by vascular smooth muscle cell [29]. Uniquely, MIF is rapidly released from preformed intracellular pools in response to LPS stimuli [30]. Consistently, in the current study, MIF secretion began to increase at 4 h after LPS stimulation, this increase sustained 24 h. Li’s research also confirmed there was a significantly higher level of MIF protein after stimulation with *E. coli* LPS for 24 h [21]. Interestingly, Li et al. also found that MIF protein level remained unchangeable in *P. gingivalis* LPS-treated reconstituted human gingival epithelia [21]. While our study showed that live *P. gingivalis* ATCC 33277-induced MIF secretion was weaker and much later than that induced by LPS. The results indicated that *P. gingivalis* ATCC 33277 had a different mechanism in inducing MIF expression compared to LPS. It has been reported that *P. gingivalis* can invade endothelial cells and remain viable for extended periods [31]. It is speculated that the invasion of *P. gingivalis* has significant repercussions for the physiological status of the cell.

Our findings identified a clue for the role of MIF in *P. gingivalis-*promoted atherosclerosis. Chuang’s research showed that MIF induced by Dengue virus infection activates endothelial cell tight junction opening, which may cause plasma leakage and leukocyte migration (extravasation), resulting in increased vascular permeability [32]. Bernhagen et al. proved that MIF concentrations increase substantially in the presence of stress, inflammation, and infection [33]. We also noticed that the MIF concentration was increased in an unhealthy periodontal environment. MIF expression was higher in the periodontal tissue of chronic periodontitis patients than in that of healthy patients [21]. In experimental gingivitis patients, MIF protein expression in the gingival crevicular fluid started increasing 1 week after the occurrence of inflammation in the 46–77 years old age group. The trend in prostaglandin E2 expression is similar to that of MIF expression. According to statistical analyses, the MIF and PGE-2 concentrations are correlated, which suggests that MIF and PGE2 interact with each other have synergistically in inflammatory conditions [34].

The role of MIF in *P. gingivalis* infection was further investigated. MIF is a multifunctional cytokine with enzymatic tautomerase activity, and its inhibitor ISO-1 can block the activity of MIF [35]. We evaluated ICAM-1 expression by performing Western blot and qRT-PCR in endothelial cells infected with *P. gingivalis* for 24 h at an MOI of 100. Our prior work found that ICAM-1 expression and monocyte-endothelial cell adhesion were increased when endothelial cells were infected with *P. gingivalis*, which is consistent with the results of others [36, 37]. In the presence of ISO-1, both the ICAM-1 protein and mRNA level induced by *P. gingivalis* infection were significantly decreased. However, ICAM-1 protein and mRNA expression levels were rescued by sufficient exogenous rMIF supplementation. We confirmed our results by cell adhesion assays. The endothelial cells were treated with ISO-1 or exogenous rMIF for 1 h before they were infected with *P. gingivalis* and then were co-cultured with monocytes. We found that monocyte adhesion to *P. gingivalis* ATCC 33277-infected endothelial cells was significantly inhibited by ISO-1. In contrast, sufficient rMIF supplementation retrieved the monocyte-endothelial cell adhesion. Recent evidence has suggested a role for endogenous MIF in the promotion of endothelial adhesion molecule expression [25]. Both Lin SG et al. [38] and Amin MA et al. [28] found that MIF up-regulated ICAM-1 expression in endothelial cells. Moreover, in MIF-deficient human umbilical vein endothelial cells, the initial steps of atherosclerosis, such as the binding of adhesion molecules on endothelial cells to their specific ligands on mononuclear cells, or monocytes in circulation rolling and attaching to the vascular wall, were be accomplished due to a lack of extracellular MIF [15–17]. Our findings provide direct evidence for the role of MIF in up-regulating ICAM-1 expression in *P. gingivalis* ATCC 33277*-*infected endothelial cells.

In summary, our study revealed that the MIF induced by *P. gingivalis* ATCC 33277 infection not only promoted ICAM-1 expression inendothelial cells but also activated monocyte-endothelial cell adhesion. We have shown that MIF is a very potent pathogenic factor in *P. gingivalis* ATCC 33277*-*induced atherosclerosis promotion. Suppressing MIF expression with an inhibitor or neutralizing antibody in individuals with manifest atherosclerosis may be a potential therapeutic intervention for treating this condition. However, the mechanisms whereby MIF facilitates endothelial adhesion molecule expression are unknown. Therefore, our future work will study the MIF receptor in *P. gingivalis-*infected endothelial cells.

## Conclusions

The experiments revealed that endothelial cell-expressed MIF participates in pro-atherosclerotic lesion formation caused by *P. gingivalis* ATCC 33277 infection. Our novel findings elucidate a more detailed pathological role of *P. gingivalis* ATCC 33277 in atherosclerosis.

## Abbreviations

CVD: Cardiovascular disease
DMEM: Dulbecco’s modified Eagle medium
*E. coli*: *Escherichia coli*
ELISA: Enzyme linked immunosorbent assay
GAPDH: Glyceraldehyde-3-phosphatedehydrogenase
ICAM-1: Intercellular adhesion molecule − 1
LPS: Lipopolysaccharide
MIF: Macrophage migration inhibitory factor
MOI: Multiplicity of infection
*P. gingivalis*: *Porphyromonas gingivalis*
qRT-PCR: Quantitative real-time polymerase chain reaction
rMIF: recombinant MIF
